# Trimethylamine Sensors Based on Au-Modified Hierarchical Porous Single-Crystalline ZnO Nanosheets

**DOI:** 10.3390/s17071478

**Published:** 2017-06-22

**Authors:** Fanli Meng, Hanxiong Zheng, Yufeng Sun, Minqiang Li, Jinhuai Liu

**Affiliations:** 1 College of Information Science and Engineering, Northeastern University, Shenyang 110819, China; mengfanli@ise.neu.edu.cn; 2 Department of Mechanical and Automotive Engineering, Anhui Polytechnic University, Wuhu 241000, China; hxzheng666@163.com; 3 Nanomaterials and Environment Detection Laboratory, Institute of Intelligent Machines, Chinese Academy of Sciences, Hefei 230031, China; mqli@iim.ac.cn (M.L.); jhliu@iim.ac.cn (J.L.)

**Keywords:** Au modification, hierarchical porous single-crystalline, trimethylamine, highly sensitive, ZnO nanosheets

## Abstract

It is of great significance for dynamic monitoring of foods in storage or during the transportation process through on-line detecting trimethylamine (TMA). Here, TMA were sensitively detected by Au-modified hierarchical porous single-crystalline ZnO nanosheets (HPSCZNs)-based sensors. The HPSCZNs were synthesized through a one-pot wet-chemical method followed by an annealing treatment. Polyethyleneimine (PEI) was used to modify the surface of the HPSCZNs, and then the PEI-modified samples were mixed with Au nanoparticles (NPs) sol solution. Electrostatic interactions drive Au nanoparticles loading onto the surface of the HPSCZNs. The Au-modified HPSCZNs were characterized by X-ray diffraction (XRD), scanning electron microscopy (SEM), transmission electron microscopy (TEM) and energy dispersive spectrum (EDS), respectively. The results show that Au-modified HPSCZNs-based sensors exhibit a high response to TMA. The linear range is from 10 to 300 ppb; while the detection limit is 10 ppb, which is the lowest value to our knowledge.

## 1. Introduction

Freshness is one of the most important attributes to define the market value for fish in the food industry [1]. Currently, there are many technologies for fish freshness determination based on visible light spectroscopy, electrical properties, image analysis, colour, electronic noses, etc. [2]. Volatile compounds measurement is one of the vital methods to determinate fish freshness. Many volatile compounds contribute to fish odor such as ammonia, trimethylamine (TMA), hydrogen sulphide, and methylmercaptan, etc. [3]. Thereinto, TMA is an effective indicator for freshness of fish which may accelerate the decomposition of trimethyl-N-oxide in fish after death. The lowest detectable concentration of TMA was just at a ppm level so far. It was reported that MoO_3_ microrods [4] and punched ZnO nanobelt network [5]-based sensors could reach a ppb level response; however, the operating temperature of them were high. Hence, it is significant to develop a highly sensitive and low operating temperature detection method toward TMA at a trace level.

Comparing to other sensing technologies, metal oxide semiconductor sensors have been widely used due to their advantages including low cost, compact size, on-line and simple operation [6,7]. During the past years, sensing metal oxide semiconductors with different morphologies and structures have been used to fabricate gas sensors, such as punched ZnO nanobelt network [5], SnO_2_-ZnO nanocomposite [8], TiO_2_-SnO_2_ nanocomposite [9], ZnO-In_2_O_3_ composite nanofibers [10], CdO-Fe_2_O_3_ nanomaterials [11], and branch-like hierarchical hetero-structure (α-Fe_2_O_3_/TiO_2_) [12]. ZnO has been used as gas-sensing materials because of its unique electronic properties [13]. It has been reported that Liu et al. [14] synthesized porous single-crystalline ZnO nanosheets which not only exhibit high gas-sensing responses, short response time and recovery time, but also possess significant long-term stability. Haiyan Song et al. [15] synthesized three kinds of porous ZnO nanostructures and found that the porous single-crystalline ZnO nanosheets showed high gas-sensing property.

As described previously, most of the sensing materials for TMA sensors are composed of ZnO with different morphologies which are doped by p-type semiconductor to improve sensing properties. Hyung-SikWoo et al. [16] prepared one-dimensional ZnO-Cr_2_O_3_ hetero-nanostructures which exhibited high selectivity and sensitivity to TMA due to the p–n junctions. Durgajanani Sivalingam et al. [17] fabricated Mn-doped ZnO thin film showing a detection limit of 5 ppm, because of the increasing of the electrical conductance due to the Mn in ZnO thin film.

Introduction of noble metals into metal oxide semiconductor sensing materials has been widely used for the enhancement of gas-sensing properties [18,19,20,21,22]. The noble metals act as catalysts for dissociation of gas molecules into more reactive species, thereby improving the gas sensitivity [23]. In our previous work, Ag and Pt have been used to decorate porous single-crystalline ZnO nanosheets and Au has been used to modify flower-like hierarchical ZnO structures, which exhibited high responses to volatile organic compounds (VOCs) [24,25,26].

Herein, combining the advantage of noble-metal modification with the hierarchical porous single-crystalline ZnO structures, the Au-modified hierarchical porous single-crystalline ZnO nanosheets (HPSCZNs) are presented. The results show that the fabricated sensors exhibit excellent sensing performance to TMA at 260 °C.

## 2. Experiment

### 2.1. Materials and Instrumentation

Zinc acetate, Urea, Chloroauric acid, Sodium citrate and Polyethyleneimine (PEI) were all purchased from Sinopharm Chemical Reagent Co., Ltd. (Shanghai, China) and analytically pure grade.

The crystal structure of the samples was determined on X-ray diffraction (XRD, Philips X’pert PRO, Eindhoven, The Netherlands) with CuKα radiation. Furthermore, the morphologies of the samples were characterized on field-emission scanning electron microscopy (FE-SEM, Sirion-200, FEI Company, Houston, TX, USA) and high-resolution transmission electron microscopy (HRTEM, JEM-2010, JEO, Tokyo, Japan). Energy dispersive spectroscopy (EDS) was measured on INCA X-Max 50 (Oxford Instruments, Oxford, UK). Zeta potential was measured on Potential analyzer Zeta Check (Horiba Scientific, Kyoto, Japan).

### 2.2. Preparation of the HPSCZNs

The HPSCZNs were synthesized by a one-pot wet-chemical method followed by an annealing treatment [27,28,29]. A typical procedure is as follows; 3.5 g of urea and 1 g of zinc acetate were dissolved into 40 mL of deionized water. After stirring for 30 min, the transparent solution was sealed in a conical flask and following a heat treatment at 95 °C for 6 h in an oven. Naturally cooling down, the white precipitate was then centrifuged and washed by deionized water and ethanol three times and then dried at 60 °C. Lastly, the HPSCZNs were obtained after annealing the precursor at 300 °C for 2 h in air in a muffle furnace.

### 2.3. Synthesis of the Au Nanoparticles (NPs) and Au-Modified HPSCZNs

Au nanoparticles (NPs) were synthesized by classical Frens method [30]. One hundred milliliters of 0.01% HAuCl_4_ was heated to boil in a three-necked flask, and then 4 mL of sodium citrate solution was added. The Au NPs were obtained when the solution’s color became red wine.

Forty milligrams of HPSCZNs were added into 80 mL of polyethyleneimine (PEI) solution (pH = 8), and then stirred for 1 h. In addition, the PEI-modified HPSCZNs were centrifuged and washed by deionized water three times and then dried at 70 °C. Finally, 40 mg of PEI-modified HPSCZNs were added into 10 mL of Au NPs sol solution and stirred for 20 min for several times (1, 2, 3, 4 and 5 times) to modify different contents of Au NPs on the HPSCZNs (#1, #2, #3, #4 and #5 samples). Finally, they were centrifuged and coated onto ceramic tube as sensing materials. The sensing materials need to be heated at 300 °C for 2 h in the muffle furnace to remove the PEI before gas-sensing measurement.

### 2.4. Gas-Sensing Measurement System and the Gas Sensor Fabrication

**Figure 1:**
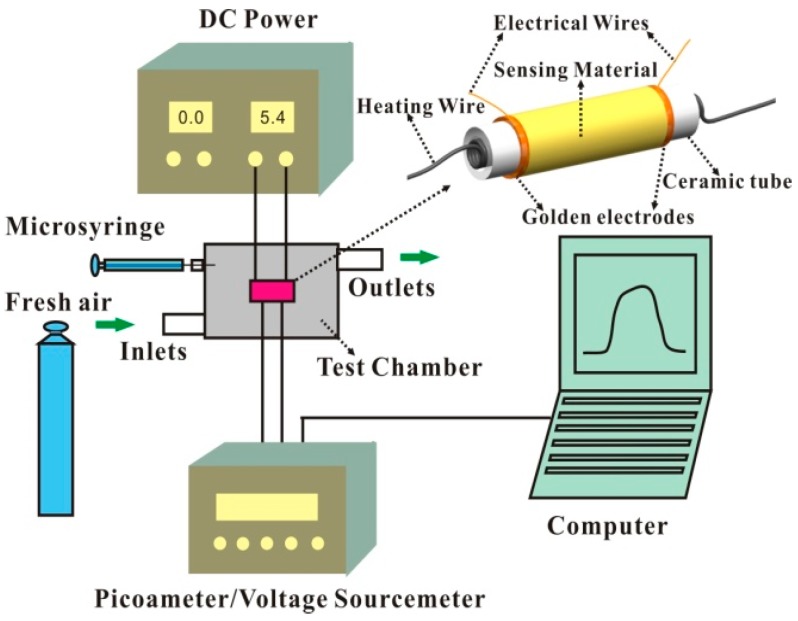
Schematic diagrams of the experimental system and the gas sensor.

The abridged general view of the gas-sensing measurement system is showed in Figure 1. The gas-sensing measurement system is installed in a square and closed test chamber with a volume of 1000 mL. The sensor is placed in the center of the test chamber. The sensing materials are coated on the ceramic tube with a pair of pre-prepared gold electrodes. A piece of nichrome wire about 32 Ω as heating wire is placed in the interior of the ceramic tube. There are inlets and outlets for gas flow. A Keithley 6487 picoameter/voltage source meter was used to record the change of current as well as to provide a power source. Applying a constant voltage of 1 V onto the electrodes between the sensing films, the current flowing through it was measured and captured. According to the gas-sensing test, a certain number of different volumes’ organic vapors (e.g., ethanol, ammonia, TMA and acetone, etc.) were introduced into the test chamber from the headspace above the liquid samples by a microsyringe [31,32]. The nominal sample concentration is calculated by the following formula:

(1) $$C=\frac{P_{0}\times V_{i}}{P_{a}\times V_{c}}$$

where *P*_0_ is the equilibrium vapor pressure at room temperature; *P*_a_ is the standard atmosphere pressure; *V*_i_ is the volume of the test gas injected by the microsyringe; *V*_c_ is the volume of the test chamber. The low concentrations of samples were prepared by diluting the vapors in a vessel before injecting them into the test chamber. At last, after injecting fresh air to release the target gas in the testing chamber, the gas-sensing measurement was finished.

The response of the sensor is defined as:

S = R_a_/R_g_ = I_g_/I_a_ (2)

In the equation, R_a_ and R_g_ are the electric resistances of the sensor in air and target gas, respectively. I_a_ and I_g_ are electric currents of the sensor which represent air and target gas, respectively.

The response time is defined as the time when the change of current reaches 90% of the balanced current on exposure to a target gas. Similarly, the recovery time is defined as the time reached a 90% reversal of the current.

## 3. Results and Discussion

### 3.1. Characterization of the Au-Modified HPSCZNs

**Figure 2:**
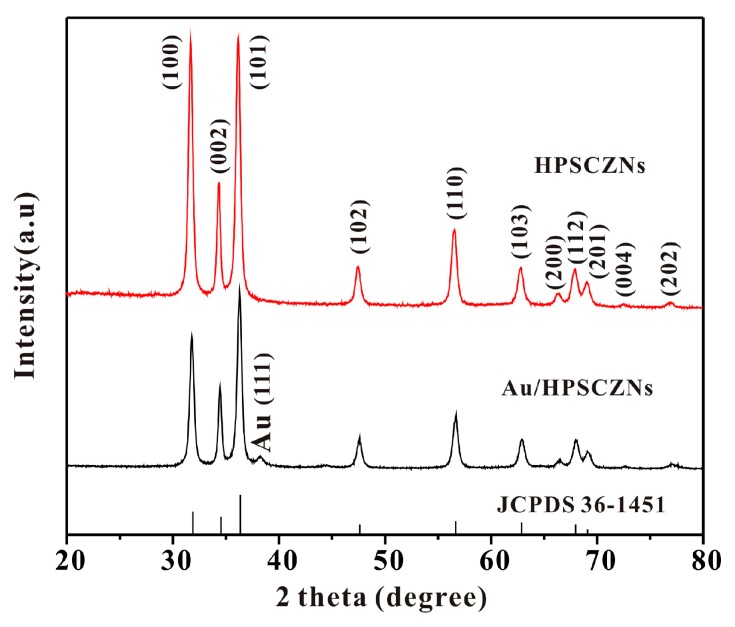
XRD patterns of the unmodified hierarchical porous single-crystalline ZnO nanosheets (HPSCZNs) (JCPDS 36-1451) and the Au-modified HPSCZNs.

Figure 2 shows XRD patterns of both the unmodified and the Au-modified HPSCZNs. All observed diffraction peaks match well with wurtzite ZnO (JCPDS 36-1451). Furthermore, as we can see, the diffraction peaks are narrow, suggesting that the ZnO crystallizes well. In addition, a weak peak of Au (111) appears in the pattern of the Au-modified HPSCZNs.

**Figure 3:**
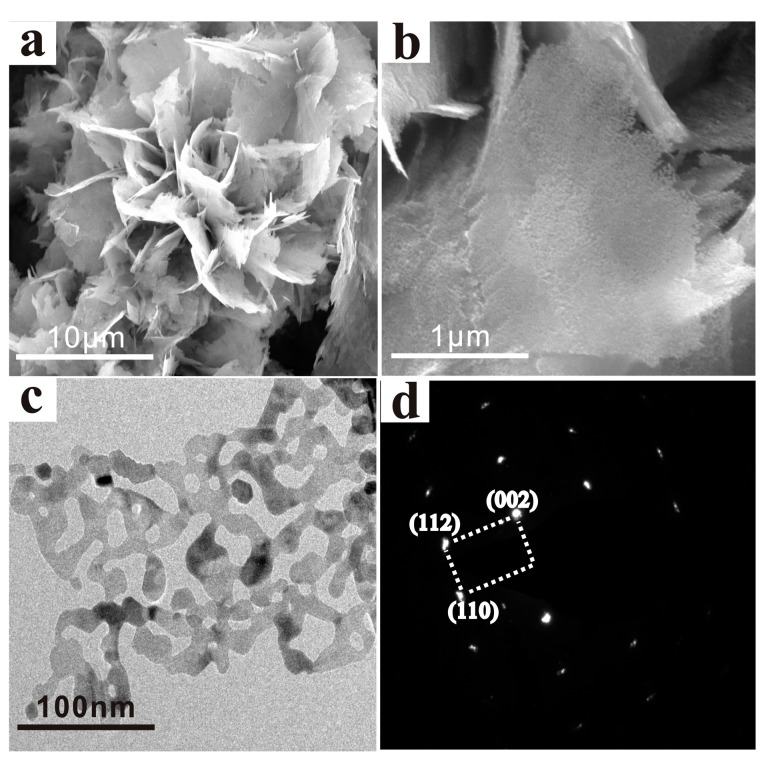
(**a**,**b**) SEM images, (**c**) TEM image and (**d**) corresponding SAED pattern of HPSCZNs.

The specific hierarchical, porous, single-crystalline structure of ZnO is further characterized by FE-SEM in Figure 3. At first, the hierarchical structure is showed in a low-magnification SEM image in Figure 3a. Many pieces of nanosheets are linked to each other, which seems like a peony with a diameter of about 15 μm. Figure 3b is a single ultra-thin nanosheet of the “flower” in a high magnification. Figure 3c,d show microstructures and corresponding selected area electron diffraction (SAED) pattern of the HPSCZNs investigated by TEM images. It can be seen that quantities of irregular nanosized mesopores randomly distribute over the single nanosheet. The latter consists of a series of well-ordered bright dots suggesting a single-crystalline structure.

**Figure 4:**
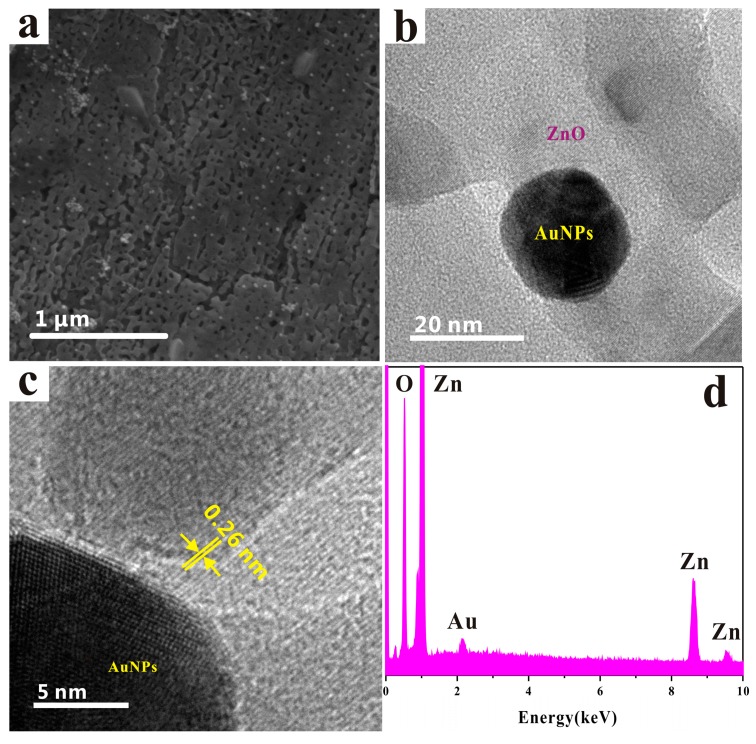
(**a**) SEM image, (**b**) TEM image, (**c**) lattice-resolved high-resolution transmission electron microscopy (HRTEM) image and (**d**) energy dispersive spectroscopy (EDS) spectrum of the Au-modified HPSCZNs.

Figure 4 shows the characteristics of the Au-modified HPSCZNs. Figure 4a shows the SEM image of a nanosheet loading with uniform Au NPs. Figure 4b,c are the TEM and HRTEM images of Au-modified HPSCZNs, which shows that Au NPs adsorb on the surface of the ZnO. The clear and coherent lattice fringes of ZnO are also observed. The lattice spacing of 0.26 nm can be indexed to the (002) planes of the hexagonal phase of ZnO. Au NPs can be combined with ZnO through the electrostatic interactions, since citric acid carries negative charges while PEI carries positive charges. Figure 4d is the EDS spectrum of Au-modified HPSCZNs, which shows that the as-synthesized porous products compose of element Zn, O and Au.

### 3.2. Formation Process of Au-Modified HPSCZNs

**Figure 5:**
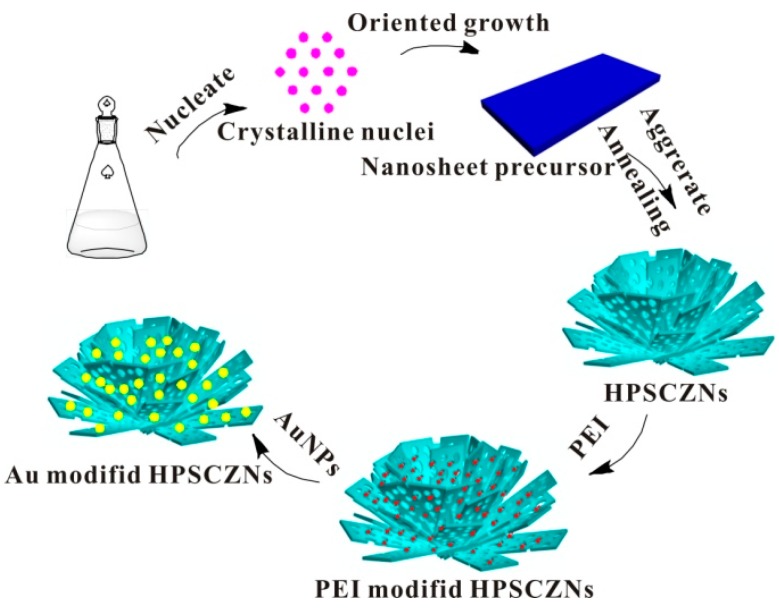
Schematic illustration of the formation process of the Au-modified HPSCZNs.

Schematic illustration of the formation process of the Au-modified HPSCZNs are shown in Figure 5. Briefly, the formation process of Au-modified HPSCZNs contains three steps: the formation of the precursor (Zn_4_CO_3_(OH)_6_·H_2_O), the annealing treatment of the precursor and the modification of Au NPs.

Firstly, crystalline nuclei form in the solution of zinc salt and urea, and then nuclei grow up via Ostwald ripening. Owing to the intrinsic anisotropic character of hexagonal structure, the aggregations would rearrange themselves and grow up along the c-axis to decrease the energy of the system during that process [33]. Chemical reactions of the precursor formation mechanism are as follows [34]:

(3) $$CO{\left(NH_{2}\right)}_{2}+3H_{2}O\to CO↑+2NH_{4}OH$$

(4) $$NH_{4}OH\to NH_{4}^{+}+{\mathrm{OH}}^{-}$$

(5) $$4Zn^{2+}+{\mathrm{CO}}_{2}+6OH^{-}+2H_{2}O\to Zn_{4}CO_{3}{\left(OH\right)}_{6}•H_{2}O+2H^{+}$$

The precursor decomposes to release CO_2_ and H_2_O under annealing at 300 °C as the following equation:

(6) $$Zn_{4}CO_{3}{\left(\mathrm{OH}\right)}_{6}H_{2}O\to 4ZnO+CO_{2}↑+4H_{2}O↑$$

**Figure 6:**
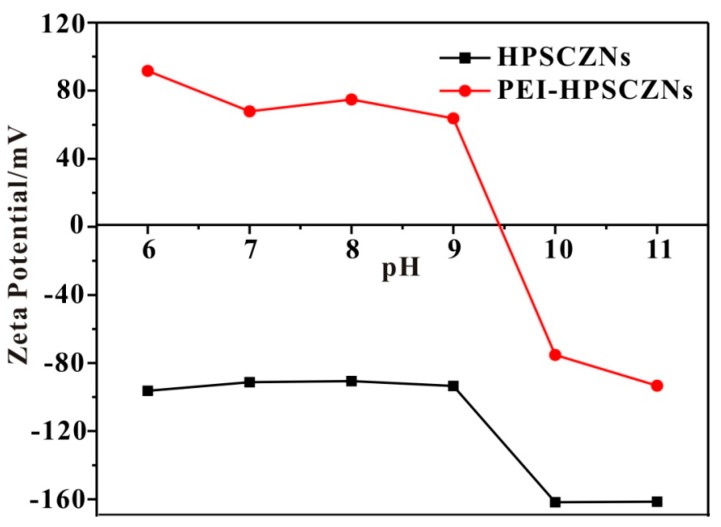
Relationship between Zeta potential of HPSCZNs and PEI-HPSCZN with different pH value.

As showed in Figure 6, Zeta potential of HPSCZNs are all negative when pH changes from 6 to 11. When modified with Au NPs, Zeta potential changes to positive as pH is lower than 9.5.

When mixing HPSCZNs with PEI aqueous solution, electrostatic interactions enable PEI loading on the surface of HPSCZNs. In this condition, surface charges of the HPSCZNs are adjusted to positive. The Au NPs are negative charged due to the citric acid on its surface.

### 3.3. Operating Temperature of Au-Modified HPSCZNs

**Figure 7:**
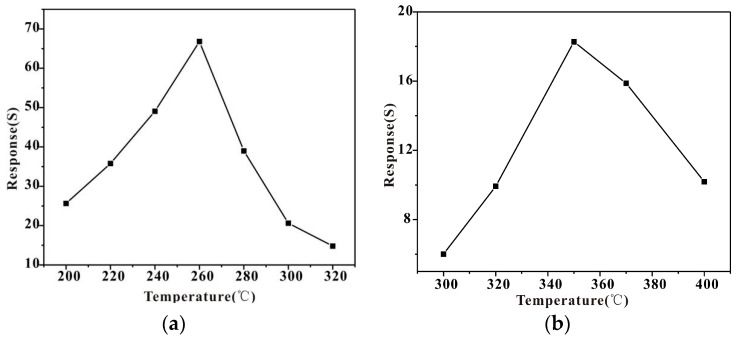
Operating temperature of (**a**) the Au-modified HPSCZNs and (**b**) the unmodified HPSCZNs-based sensors to 30 ppm of trimethylamine (TMA).

Reactions between target gases and the adsorbed oxygen species on material surface is related to the activity of the adsorbed oxygen species. At different temperatures, there are two types of adsorbed oxygen species, one is molecular ion (O_2_^−^), another is atomic ions (O^−^, O^2−^). The atomic form dominates above 150 °C, while molecular species work at lower temperature [35]. Figure 7 shows the plots of the response versus working temperature of the Au-modified HPSCZNs and the unmodified HPSCZNs-based sensors to 30 ppm of TMA. As we can see, the Au-modified HPSCZNs sensor exhibits different responses as shown in Figure 7a. The sensitivity is highest at 260 °C, which is lower than the unmodified HPSCZNs sensor showed in Figure 7b, which is caused by the Au modification [36].

### 3.4. Gas-Sensing Property of Different Contents of Au NPs-Modified HPSCZNs

**Figure 8:**
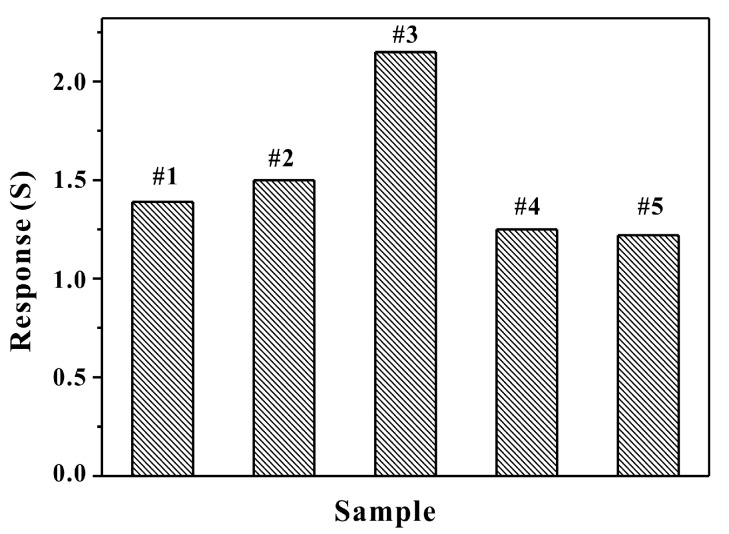
Response curves of the HPSCZNs sensor with different contents of Au NPs to 100 ppb of TMA.

Figure 8 shows the response curves of HPSCZNs with different contents of Au NPs modification to 100 ppb of TMA. In the modification process, PEI-modified HPSCZNs were centrifuged and washed by deionized water. If content of PEI was too high, Au NPs would aggregate and then the improvement of sensing properties would be poor. Therefore, the content of PEI is also needed to be proper. The greatest factor that influences sensitivity is the contents of Au NPs. From the responses, it can be observed that sample #3 possesses the highest response, which means that there are times that Au NPs modification is optimal in the synthesis process. That is because the Au NPs could aggregate together when the Au NPs modification is overdosed, which makes the response reduce.

### 3.5. Comparison of Unmodified and Au-Modified HPSCZNs Sensors to TMA

**Figure 9:**
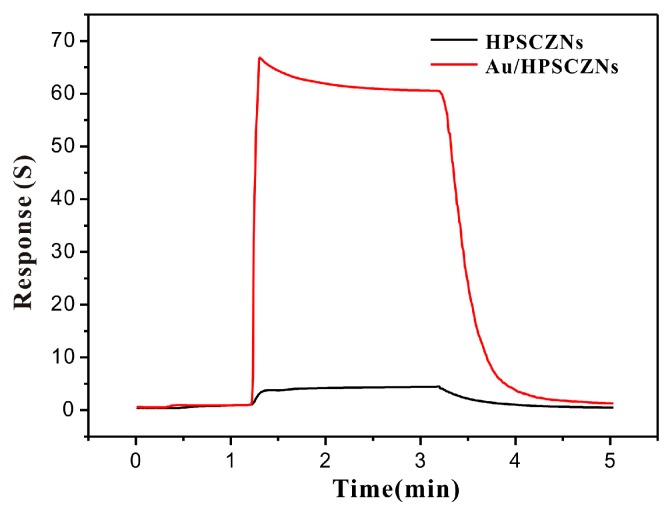
Response curves of the unmodified and the Au-modified HPSCZNs sensors to 30 ppm of TMA at 260 °C.

In order to investigate gas properties of unmodified and Au-modified HPSCZNs sensors, response curves of them to TMA is shown in Figure 9. It is clear that the response curve of the Au-modified HPSCZNs sensor is higher than that of the unmodified HPSCZNs sensor to 30 ppm TMA. The response is 5.9 and the response and recovery times of them are 21 s and 70.5 s, respectively. The response of the modified one is 65.8 and the response and recovery times are 3.3 s and 64 s, respectively. The Au-modified HPSCZNs sensor processes higher response and shorter response and recovery times compared to the one without modification, which might be relevant to modification of Au NPs.

### 3.6. Gas-Sensing Property of the Au-Modified HPSCZNs Sensor to TMA

**Figure 10:**
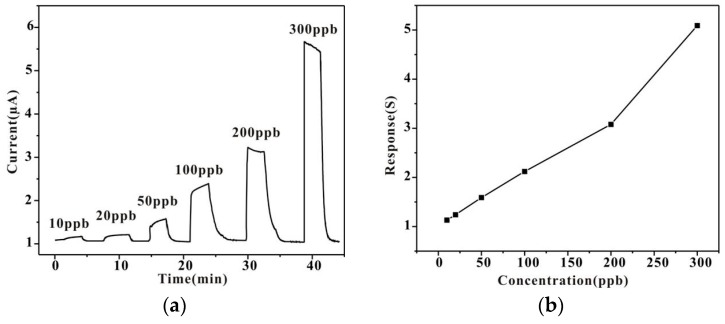
(**a**) Responses of the Au-modified HPSCZNs structures to different concentrations of TMA and (**b**) the corresponding calibration curve.

The responses of the Au-modified HPSCZNs to TMA are shown in Figure 10. Figure 10a shows the real-time response curve of the sensor to different concentrations of TMA. Obviously, the responses of the sensor increase with TMA concentration. At the beginning, the adsorption reaction dominates the dynamic process and desorption interaction appears gradually and tends to be stable ultimately. The response linearly increases in the range from 10 to 300 ppb as shown in Figure 10b. The lowest detection concentration is 10 ppb, which is an extremely low level to our knowledge.

### 3.7. Selectivity and Stability of the Au-Modified HPSCZNs Sensors

**Figure 11:**
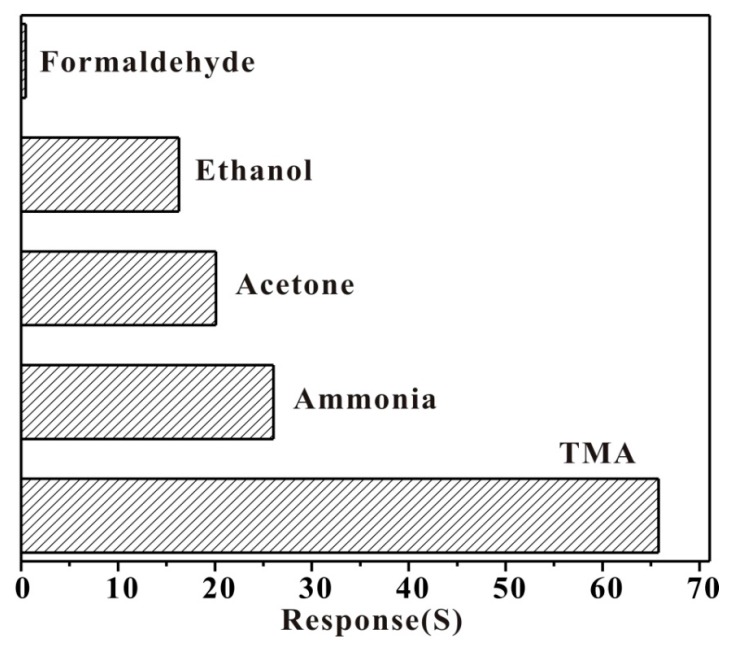
Response comparison of 30 ppm of TMA and 30 ppm of other volatile organic compounds (VOCs) at 260 °C.

It was reported that ZnO structure gas sensors showed response to many gases: CH_3_COCH_3_, CO, C_2_H_5_OH, H_2_S, HCHO, etc. [37]. Nevertheless, response comparison of 30 ppm TMA and other gases at 260 °C suggest that the Au-modified HPSCZNs sensor have high selectivity to TMA as shown in Figure 11. Response of TMA is two times higher than that of ammonia. The reactivity of the reducing gases determines the sensor’s sensitivity, so the mechanism of high selectivity to TMA can be explained from the bond energy. The molecular structures and the bond energies of many gases are showed in Table 1 [38]. The bond energy of H–CH_2_ with the highest response is only 380.7 KJ/mol, which is smaller than any other organic gases in the table. Therefore, H–CH_2_ can be easily broken to take part in adsorption reaction with sensing material; all of this results in the high selectivity of TMA.

**Figure 12:**
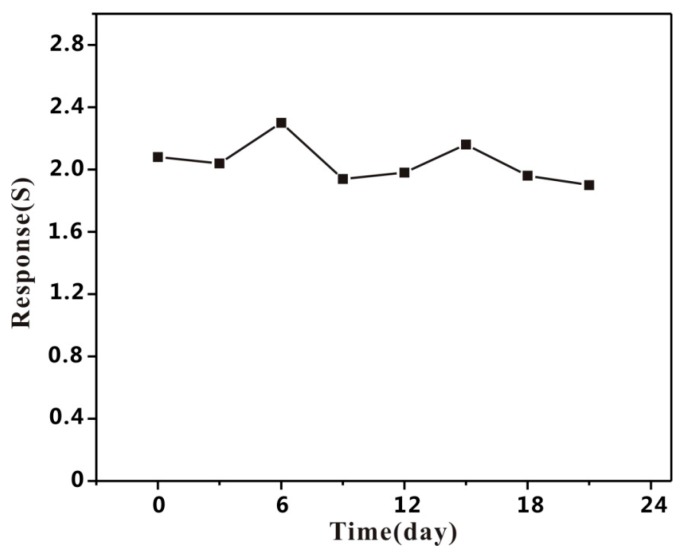
Stability of the Au-modified HPSCZNs sensor to 100 ppb of TMA.

Stability has been investigated by the gas response of Au-modified HPSCZNs sensors to 100 ppb of TMA within 21 days as shown in Figure 12. During the experimental process, the response of the Au-modified HPSCZNs sensor hardly floats up and down, indicating that the Au-modified HPSCZNs sensors exhibit a good stability.

### 3.8. Sensing Mechanism of Au-Modified HPSCZNs

The Au-modified HPSCZNs sensor becomes more sensitive due to the mesopores on each nanosheet, hierarchical morphology, single-crystalline structure and Au NPs modification. On one hand, mesopores enlarge specific surface area of HPSCZNs and offer plenty of active sites for surface chemical reactions; on the other hand, hierarchical structure enables gases to diffuse into the film and the inner of the material by molecular diffusion [39,40,41]. Given as a Knudsen flow, the diffusion coefficient can be defined by the following equation [42]:

(7) $$D_{K}=\frac{\varepsilon d}{3\tau }{\left(\frac{8RT}{\pi M}\right)}^{\frac{1}{2}}$$

where D_k_, ε, d, τ, R, T and M are Knudsen diffusion coefficients, dimensionless porosity, dimensionless tortuosity, pore diameter, gas constant, temperature and molar mass, respectively. Obviously, the gas diffusion is proportional to pore diameter and porosity presented in the equation, the pore diameter of HPSCZNs is around 30 nm; smaller and less overlapped necks interact with test gases, which results from a greater porosity [43]. On the basis of these two advantages, gas molecules are permitted to diffuse into the film rapidly. What is more, single-crystalline structure also plays a significant role for improving sensitivity. As is known to all, electrons need to overcome boundary barrier between particles when they transport from one side to another; it would obviously decrease the sensitivity. The as-prepared products that contain lots of single-crystalline ZnO nanosheet structures that comply with the grain boundary barrier model are equivalent to reduce particle boundary barriers, and further increase the sensitivity and stability. Lastly, Au NPs modification acts as the most critical role to improve the sensitivity. It not only possesses high catalytic activity but also increases the depth of the space-charge layers [44]. When the sensor exposes to air, Au NPs accelerate O_2_ which is adsorbed on the surface of HPSCZNs capturing electrons to form O_2_^−^, O^−^ and O^2−^ ions.

## 4. Conclusions

In conclusion, HPSCZNs were successfully synthesized. Under the optimal operating temperature of 260 °C, the Au-modified HPSCZNs sensor exhibits a high response to TMA. The linear range is 10–300 ppb; while the lowest detection is 10 ppb. The Au-modified HPSCZNs, combining with the advantages of high response, long-term stability, low power consumption and cost, may provide a novel pathway to make gas sensors for the detection of the freshness of fish and some other foods.

## Figures and Tables

**Table 1:** Properties of gas molecules.

Gas Type	Molecular Structures	Bond	Bond Energy (KJ/mol)
Formaldehyde	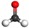	H–CHO	436
Ethanol	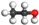	H–OCH_2_CH_3_	436
		H–CH_2_	473
		H–CH	452
Acetone	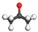	H–CH_2_OCH_3_	393
Ammonia	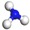	H–NH_2_	435
TMA	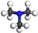	H–CH_2_	380.7
